# The impact of adding dual and triple combinations of quicklime and plastic wastes and palm fibers on the California bearing ratio of fine sand

**DOI:** 10.1038/s41598-025-09647-y

**Published:** 2025-07-08

**Authors:** Ahmed H. Elbosraty, Mohamed A. Bahr, Ahmed M. Ebid

**Affiliations:** 1 Department of Civil Engineering, Al-Azhar University in Egypt, Cairo, Egypt; 2 Department of Structural Engineering and Construction Management, Future University in Egypt, New Cairo, Egypt

**Keywords:** California bearing ratio, Fine sand, Quicklime, Palm fiber, Plastic wastes, Civil engineering, Mechanical engineering

## Abstract

Fine sand, widely distributed across arid and semi-arid regions, presents challenges due to its low bearing capacity and susceptibility to deformation. This study investigates the enhancement of the California Bearing Ratio (CBR) value of fine sand through the incorporation of palm fibers, plastic waste, and quicklime. This study investigates the enhancement of the CBR of fine sand using palm fibers, plastic waste, and quicklime. Through two experimental phases, optimal dosages were determined as 5.0% quicklime, 0.75% plastic waste, and 1.0% palm fiber. Single additive treatments yielded CBR improvements of 195%, 125%, and 275%, respectively. Combinations revealed that mixing quicklime with palm fibers decreased enhancement efficiency due to chemical incompatibility. Notably, palm fiber–plastic waste mixtures proved more sustainable. This research offers a cost-effective, eco-friendly solution for improving subgrade conditions with clear quantitative outcomes. The findings underscore the potential of recycling plastic waste by mixing with palm fibers for sustainable improvement of fine sand properties. By reducing plastic pollution and encouraging circular resource usage, the combination of natural palm fibers and recovered plastic waste enhances soil performance and promotes environmental sustainability. Furthermore, this approach is more affordable than traditional soil stabilizing methods, especially in areas where local resources are easily accessible.

## Introduction

The enhancement of soil engineering residences is a critical vital in civil engineering, in particular whilst addressing the task of constructing systems on complicated soil substrates. Among these substrates, fine sand is a prevalent and formidable type, dispersed across various geographical regions but liable to collapse underneath implemented loads. Different ways to improve soil have been created to address these problems, including mechanical methods like compaction and confinement, and chemical stabilization with additives that increase strength and durability^1–4^. In recent years, the pursuit of effective soil improvement strategies has brought about improved exploration and alertness of various materials to enhance soil stability^5^. Among chemical stabilizers, lime has long been recognized for its efficacy in enhancing the engineering properties of a wide range of soils. Abbasi and Mahdieh^6^ demonstrated that the addition of lime and natural pozzolan to silty sand soils can significantly increase the California Bearing Ratio (CBR), particularly under optimal moisture content conditions. Similarly, Al-Rawas et al.^7^ reported improvements in the swelling characteristics and plasticity of expansive soils treated with lime and cement. Baghdadi and Rahman^8^ evaluated the stabilization of dune sand using cement kiln dust, noting a significant enhancement in both compressive strength and CBR values.

Ji-ru and Xing^9^ studied the impact of lime and fly ash on the geotechnical properties of expansive soil, both individually and in combination. Their experiments revealed that “Maximum Dry Density” MDD, “Free Swell” FS, and swelling capacity under 50 kPa pressure decreased with growing quantities of lime and fly ash, while the CBR value, optimum moisture content, and percentage of coarse particles increased. The study investigated the impact of aggregate gradation on resilient modulus and CBR values for both recycled and quarried aggregates. The consequences offer important insights into sustainable material utilization, underscoring the look at’s recognition on integrating recovered plastic waste to enhance subgrade overall performance. The results contribute to the developing body of research advocating environmentally friendly pavement substances.

Recent studies have also explored the reinforcement of soils with natural and synthetic fibers. Elbosraty et al.^10^ examined the inclusion of palm fibers in fine sand and found a substantial improvement in the CBR values due to mechanical interlocking effects. Yusof et al.^11^ combined palm fibers with hydrated lime and observed enhanced permeability reduction and mechanical strength in stabilized sandy soils. Azadegan et al.^12^ investigated the reinforcement of clay with palm fibers and highlighted that while such fibers can enhance mechanical properties, their performance can vary depending on environmental conditions and soil type. In the pursuit of sustainable construction practices, the utilization of waste materials such as recycled plastics has gained attention. Kumar et al.^13^ demonstrated that the incorporation of plastic strips into clayey soils can significantly improve CBR values when optimal strip length and dosage are used. Alzaidy^14^ explored the combined use of eggshell powder and plastic waste strips for soil stabilization, reporting enhanced unconfined compressive strength and CBR performance. However, the environmental impact of traditional stabilizers such as lime cannot be overlooked. Lime production is energy-intensive and contributes to CO₂ emissions, while its application can affect soil microbiota^15^. In contrast, palm fibers, being biodegradable, and recycled plastic wastes offer more environmentally friendly alternatives for soil stabilization^16^. The CBR test remains a cornerstone method in evaluating the load-bearing capacity of stabilized soils. Originally developed in the 1930s by the California Division of Highways, the CBR test provides a standardized approach for assessing subgrade and base course materials for pavement and infrastructure projects^17,18^. While the use of single additives like lime, palm fibers, or plastic waste has been widely studied, limited research has examined the combined effects of these materials. Particularly, the interaction between quicklime and natural fibers such as palm fibers needs further investigation due to potential chemical incompatibility. Alkaline environments can degrade the organic components of natural fibers, as shown by Taallah and Guettala^19^, who reported that alkali-treated palm fibers exhibited reduced reinforcement efficiency in compressed earth blocks.

Generally, adding fibers (or strips) to loose sand improves its shear strength regardless of the material used. The enhancing mechanism depends on using the tensile strength of the fibers to reinforce the sand (the fibers or strips acts as dowels across any possible shear failure surface). Accordingly, the CBR value enhances due to the improving of the shear strength of the mixture. The efficiency of the used fibers or strips depends on their tensile strength and the interaction with the sand particles (the force transferred between the sand and the fibers). The more large specific surface area and surface roughness of the fibers, the more efficient interaction force transfer with sand. Based on that, long, rough, and small diameter fibers such as palm fibers are more efficient than short, smooth and wide surface strips like plastic waste strips. On the other hand, adding lime enhances the shear strength (and accordingly the CBR) in a different mechanism. It acts as bonding agent that glues the sand particles together; hence, it provides an additional cohesion strength to the existing friction strength of the granular loose sand and hence increasing the total shear strength of the mixture.

To enhance the connection between material qualities and observed outcomes, we further elaborated on the molecular mechanisms of the stabilizing agents. Quicklime (CaO), when introduction to damp soil, hydrates to produce calcium hydroxide (Ca (OH)^₂^), which interacts with silica and alumina in the soil to generate cementitious compounds, hence augmenting strength. Palm fibers, mostly consisting of cellulose, lignin, and hemicellulose, enhance mechanical interlock and augment shear strength. Nevertheless, exposure to alkaline conditions (from lime) can deteriorate these organic structures, diminishing their efficacy, a phenomenon investigated further in our microscopic investigations. Recent advancements in sustainable soil stabilization have emphasized the pozzolanic reactions involving industrial and agricultural waste materials. Lime, when combined with agricultural products, can form additional cementitious compounds, enhance mechanical strength while reduce environmental impacts. Studies such as Olaiya et al.^20^ explored the interaction mechanisms of lime with organic residues, highlighting the potential of synergistic pozzolanic reactions to boost soil stabilization. Furthermore, Olaiya et al.^21^ and Olaiya et al.^22^ investigated the physicochemical behaviors of soil stabilized with agricultural wastes and lime blends, reporting notable improvements in strength and durability under variable environmental conditions. The effects of environmental variables on the additives were extensively studied in earlier researches. The impact of environmental variables on the non-organic additives such as plastic wastes and lime are insignificant. In fact, the high durability of plastic wastes is an environmental problem. On the other hand, the natural (untreated) organic additives such as palm fibers are not durable; they decompose after a certain period that depends on type of fibers. This decomposition is an environmental advantage and engineering disadvantage in the same time^23,24^. Building on these findings, this study aims to experimentally investigate the individual and combined effects of quicklime, palm fibers, and plastic waste on the CBR value of fine sand. By identifying optimal dosages and analyzing interaction effects, this research contributes to the development of more sustainable and efficient soil stabilization techniques suitable for resource-constrained and environmentally sensitive regions.

## Methodology

The methodology employed in this investigation started with collecting, sorting and summarizing the previously published related research works. Based on that, the doses of the used materials were determined to be used in the experimental program. The first phase of this program included ten samples, one control and nine mixtures (three additives x three doses). During this phase, the required materials (fine sand, palm fiber, plastic waste, and quicklime powder) were collected, prepared, mixed and tested to determine the compaction properties of each sample. Finally, the CBR value of each mix was measured. The aim of this phase was to determine the optimum dose for each single additive.

**Fig. 1:**
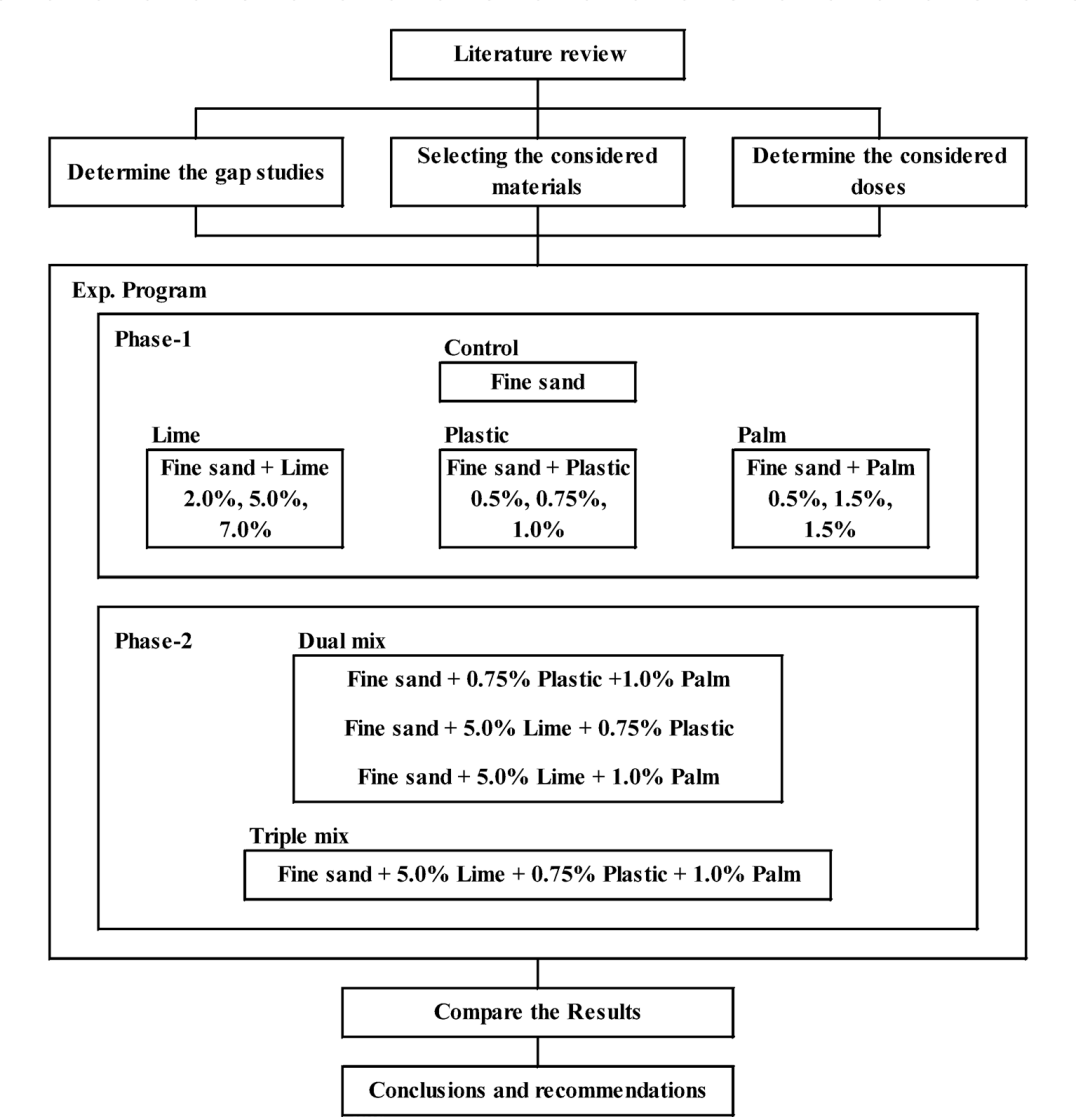
Research methodology.

The second phase of the experimental program aimed to investigate the impact of using two or more additives together on the CBR value of the mixture. Accordingly, additional four samples were prepared using the pre-determined optimum doses for each additive. Three samples for dual additives mixtures and one sample for triple additives mixture. All the results were analyzed and discussed to extract the conclusions. Figure 1 graphically presents the considered methodology.

### Collecting and preparation of the materials

**Fig. 2:**
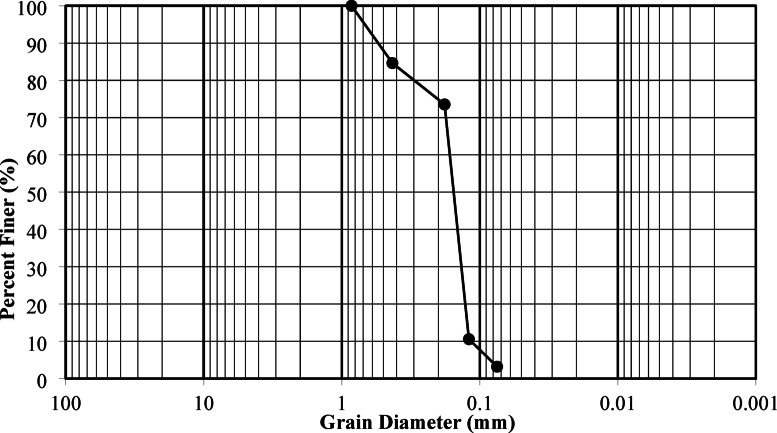
The gradation curve of the studied fine sand.

The primary materials employed in this study included fine sandy soil samples, palm fiber, plastic waste, and quicklime powder. The fine sand sample was sourced from the AL-Dabaa corridor, a desert region in Egypt. The index properties of the soil sample are detailed in Table 1, and the grading curve of the soil is illustrated in Fig. 2. Natural palm fibers, sourced from a palm plantation in Egypt, were cut to a length of 4 cm for use in this research. Quicklime was obtained from a local Egyptian vendor. Additionally, empty plastic bottles were cut into pieces of random lengths.

The chemical analysis of plastic waste is too difficult because it was a mix of different types of plastic bottles, cans and parts; on the other hand, palm fibers are manly composed of cellulose, while the lime is calcium oxide.

**Table 1:** Properties of the considered fine sand.

Soil classification	Specific gravity (Gs)	Effective diameter (D50) (mm)	Uniformity coefficient (Cu)	Coefficient of curvature (Cc)	Optimum moisture content (%)	Maximum dry density (kN/m^3^)
Fine sand	2.66	0.12	1.54	0.95	10.29	16.46

### Mixing and pre-tests

To investigate the effects of varying contents of palm fiber, plastic waste, and quicklime on the CBR value, thirteen sample with different materials and weight ratios were mixed, as shown in Table 2, as follows:

- Fine sand (Control).
- Fine sand + Quicklime (2%, 5%, and 7%),
- Fine sand + Plastic waste (0.5%, 0.75%, and 1.0%),
- Fine sand + Palm fiber (0.5%, 1.0%, and 1.5%),
- Fine sand + 0.75% Plastic waste + 1% Palm fibers.
- Fine sand + 0.75% Plastic waste + 5% Quicklime.
- Fine sand + 5% Quicklime + 1% Palm fibers.
- Fine sand + 5% Quicklime + 1% Palm fibers + 0.75% Plastic waste.

**Table 2:** Composition and additive dosages of fine sand stabilization mixes.

Sample ID	Composition	Additive percentages by weight
S1	Control (Fine Sand Only)	0%
S2	Fine Sand + Quicklime	2%
S3	Fine Sand + Quicklime	5%
S4	Fine Sand + Quicklime	7%
S5	Fine Sand + Plastic Waste	0.50%
S6	Fine Sand + Plastic Waste	0.75%
S7	Fine Sand + Plastic Waste	1.00%
S8	Fine Sand + Palm Fiber	0.50%
S9	Fine Sand + Palm Fiber	1.00%
S10	Fine Sand + Palm Fiber	1.50%
S11	Fine Sand + Palm + Plastic	1.0% + 0.75%
S12	Fine Sand + Lime + Palm	5.0% + 1.0%
S13	Fine Sand + Lime + Plastic	5.0% + 0.75%
S14	Fine Sand + Lime + Palm + Plastic	5.0% + 1.0% + 0.75%

Modified Proctor compaction tests were conducted in accordance with AASHTO T180 and ASTM D1557 standards. The CBR tests were performed following AASHTO T193 and ASTM D1883 protocols to ensure consistency and reproducibility in line with international geotechnical testing standards.

### CBR tests

CBR tests were conducted for all samples acceding to (AASHTO T-180). Samples with OMC were placed into molds in five uniform layers and compacted using 56 blows from standard hammer. The specimens were subsequently immersed in water for two days to allow for the curing process. The CBR tests were conducted in accordance with AASHTO T180/ASTM D1557 standards, following a curing period of two days at the optimum moisture content, as shown in Table 3.

**Table 3:** The recorded OMC for each sample.

Soil (control sample)	Soil + lime (2.0,5.0, 7.0%)	Soil + plastic (0.5, 0.75, 1.0%)	Soil + palm fiber (0.5, 1.0, 1.5%),	Soil + plastic + palm	Soil + lime + plastic	Soil + lime + palm	Soil + lime+ plastic + palm
10.3%	14.3%	10.3%	10.3%	10.3%	14.25%	14.15%	14.2%

## Results and discussion

### Phase-1

**Fig. 3:**
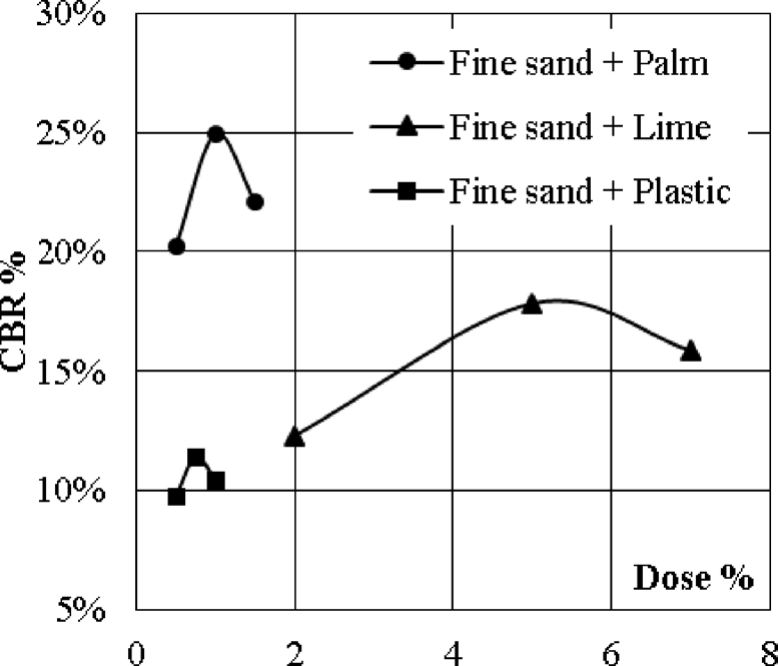
The results of the 1st phase (single additive).

The results of the first phase of the experimental program (10 samples) showed that the optimum doses were (5.0%, 0.75% and 1.0%) for lime, plastic and palm fibers respectively. Plastic mixture showed the lowest CBR values (9.7–11.4%), lime mixture showed better results (12.3–17.8%), while palm fibers mixtures were the best (20.2–24.9%). The results of this phase are graphically presented in Fig. 3.

### Phase-2

**Fig. 4:**
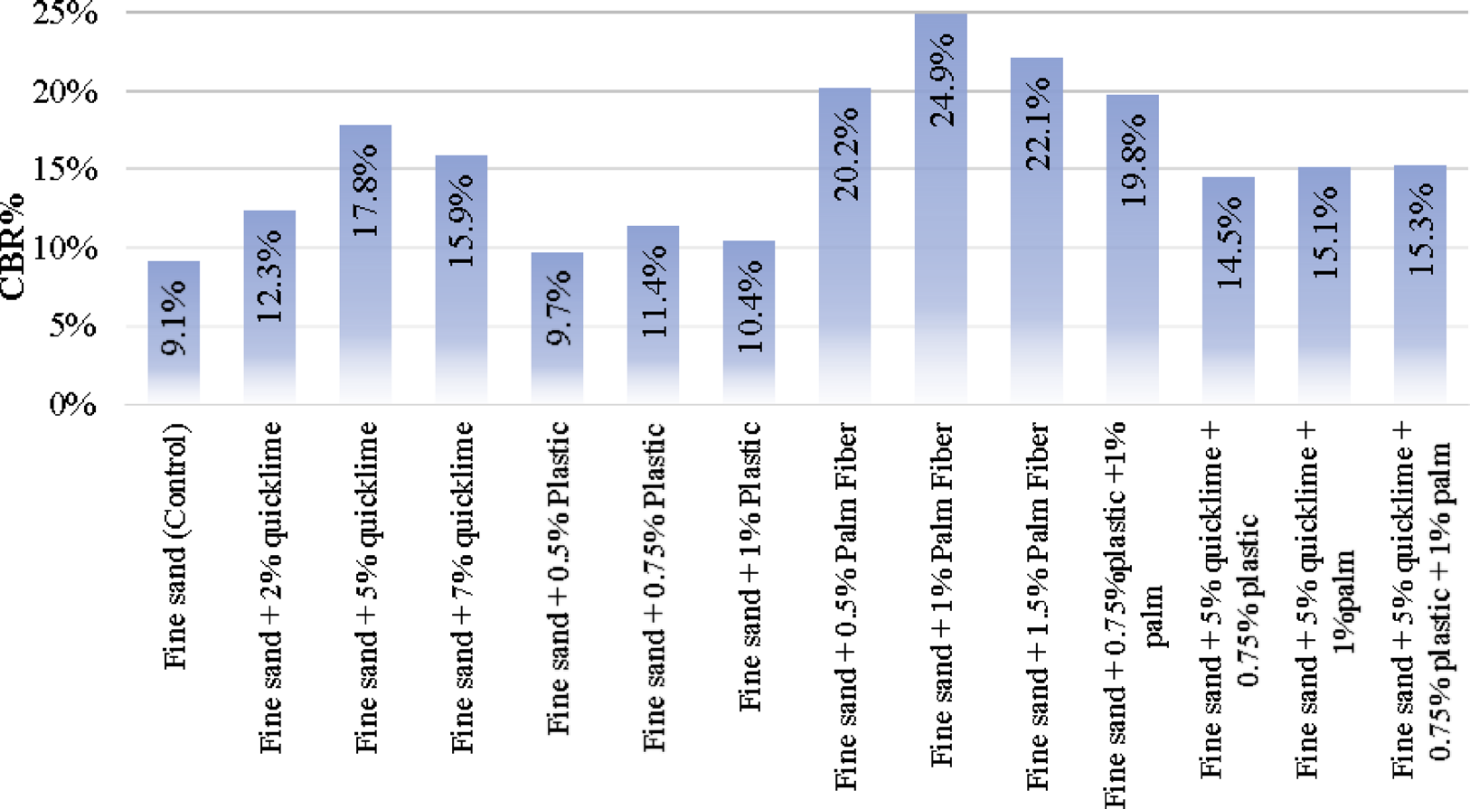
All the results of the experimental program.

The CBR values of the four samples that were mixed using the pre-determined optimum doses of each additive were (19.8%, 14.5%, 15.1% and 15.3%) for (plastic-palm, lime-plastic, lime-palm and lime-plastic-palm) respectively. All the results of the experimental program are listed in Table 4 and graphically presented in Fig. 4. Error bars are not included due to single test per sample, which is a limitation to be addressed in future studies.

**Table 4:** The values of CBR and O.M.C. for all specimens.

Sample	CBR %
Fine sand (Control)	9.1%
Fine sand + 2% quicklime	12.3%
Fine sand + 5% quicklime	17.8%
Fine sand + 7% quicklime	15.9%
Fine sand + 0.5% Plastic	9.7%
Fine sand + 0.75% Plastic	11.4%
Fine sand + 1% Plastic	10.4%
Fine sand + 0.5% Palm Fiber	20.2%
Fine sand + 1% Palm Fiber	24.9%
Fine sand + 1.5% Palm Fiber	22.1%
Fine sand + 0.75%plastic + 1% palm	19.8%
Fine sand + 5% quicklime + 0.75% plastic	14.5%
Fine sand + 5% quicklime + 1%palm	15.1%
Fine sand + 5% quicklime + 0.75% plastic + 1% palm	15.3%

**Fig. 5:**
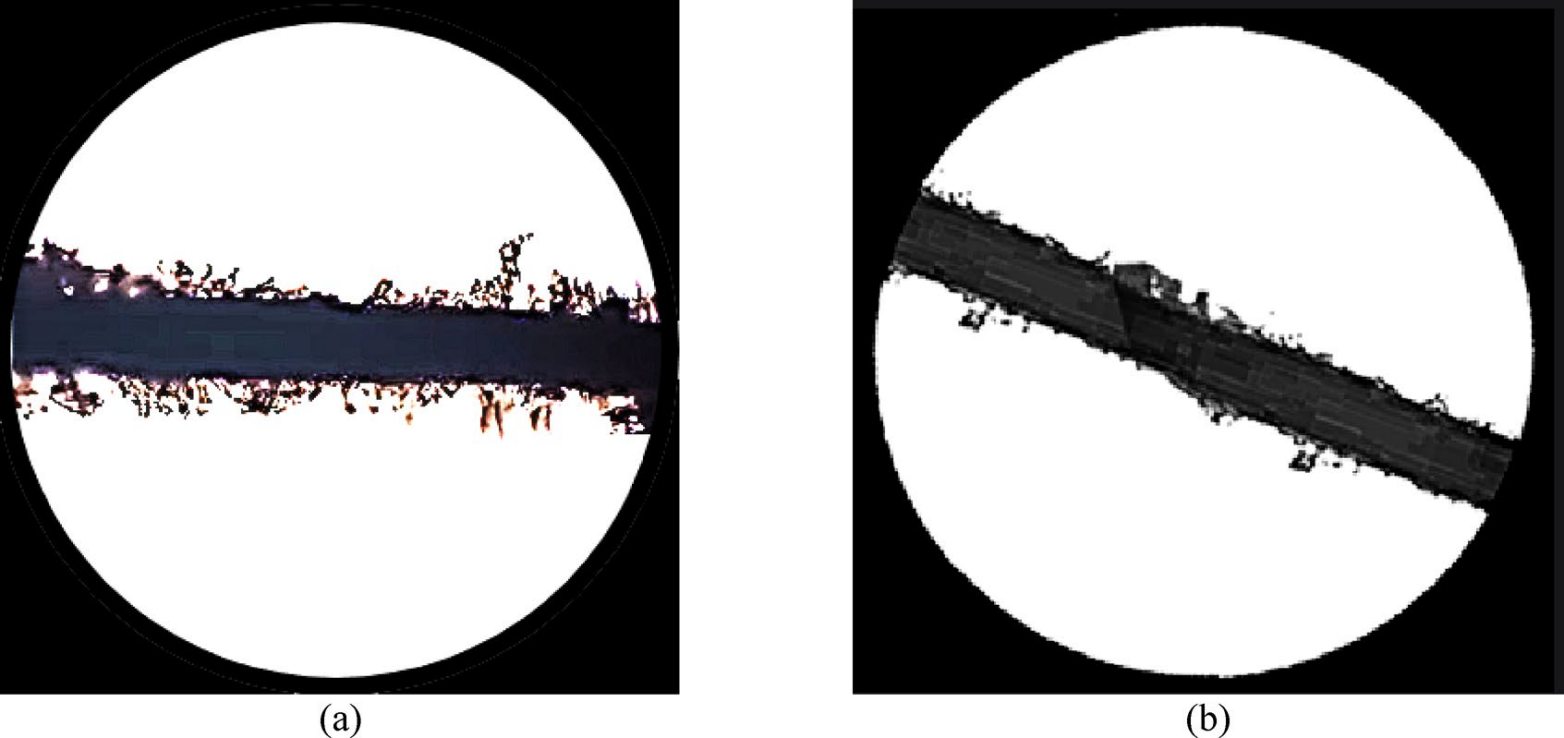
Microscopic photos for palm fiber (**a**) original Fiber, (**b**) after treating with quick lime.

Figure 5 shows two microscopic images. In Fig. 5a, untreated palm fibers appear rough with visible fibrils that contribute to mechanical interlock with fine sand. In contrast, Fig. 5b shows palm fibers after treatment with quicklime, where the surface becomes smooth, and lateral branches are lost reducing interfacial friction and reinforcement efficiency.

### Discussion

The measured OMC indicated using palm fibers and plastic wastes did not affect the OMC, this is because of their small dose (≈1.0%) and because they do not absorb water. On the other hand, the OMC increased by (14.3/10.3 = 140%) by adding quicklime because of its large dose (7.0%) and its ability to absorb significant amount of water.

Analyzing the results of the first phase indicated that using the optimum dose of palm fibers (1.0%) increased the CBR value of the fine sand by (24.9/9.1 = 275%), mixing the fine sand with 5.0% quicklime enhanced the CBR value by (17.8/9.1 = 195%). Finally, adding 0.75% by weight plastic waste to the fine sand improved the CBR value by (11.4/9.1 = 125%). These results are in good agreement with the outcomes of Abbasi^6^ for lime, Sarbaz^10^ for palm fibers and Kumar^13^ for plastic wastes.

Despite the significantly higher tensile strength of plastic waste compared to palm fibers, its smooth surface and limited flexibility reduce its interfacial strength with fine sand, which made the palm fiber more efficient.

The second phase results showed that mixing lime with palm fibers increased the CBR value by (15.1/9.1 = 165%) which is less than the enhancement of both of them separately. While mixing lime with plastic waste enhanced the CBR value by (14.5/9.1 = 160%) which equals to the average enhancement of both of them separately (195 + 125/2 = 160). Moreover, mixing the palm fiber with plastic wastes improved the CBR values by (19.8/9.1 = 220%) which is almost the average value of the improvement of them separately (275 + 125/2 = 200). Finally, mixing all the additives together increased the CBR value by (15.3/9.1 = 170%) which is less than the average of their enhancement separately (275 + 195 + 125/3 = 200).

The enhancement of the loose sand CBR due to adding (Lime + Plastic wastes) or (Plastic wastes + Palm fibers) was almost the average of the enhancement values of each individual additive, which indicted that there is no interaction between plastic wastes and lime or palm fibers. On the other hand, the improvement in the CBR due to (Lime + palm fibers + plastic wastes) is less than the average improvement of the individual additive. In addition, the improvement due to adding (lime + palm fibers) was lower than the improvement of each individual additives themselves (not even the average), which clearly indicated the negative impact of combining lime with palm fibers on both of them.

In order to investigate this point, a sample of the palm fiber was photographed under microscope then it was treated with quick lime solution for 24 h and then it was photographed again. Comparing the photos in Fig. 5 showed that the original fiber was rough and have lateral branches along their length, while the treated one became smooth and lost its lateral branches, accordingly, treating the palm fibers with quick lime reduces the interaction force between them and the surrounding sand particles. On the other hand, the part of the lime that reacted with the palm fiber will not act as bonding agent to glue the sand particles; hence, the efficacy of the lime was also decreased. Figure 5 clearly illustrates the change in surface morphology of palm fibers before and after treatment with quicklime. Untreated fibers (Fig. 5a) exhibit a coarse texture and visible fibrils that enhance mechanical interlock with sand particles. However, after immersion in a lime solution, the fibers become smoother and lose their lateral branches (Fig. 5b), significantly reducing the contact surface area and frictional resistance. This deterioration in surface texture explains the reduction in reinforcement effectiveness when palm fibers are mixed with lime.

It could be noted from the previous analysis that mixing the quicklime with the palm fiber decrease the enhancement efficiency of both of them. This is because of the harmful effect of quicklime on natural organic matter such as palm fibers. From an environmental perspective, the use of palm fibers and recycled plastic waste offers more sustainable alternatives compared to quicklime. While palm fibers are biodegradable, plastic waste stabilization contributes to waste recycling efforts. However, it is important to note that the long-term durability of natural fibers remains a concern, as they are susceptible to biodegradation under field conditions, as highlighted by previous studies^23,24^. Quicklime, although effective, has environmental drawbacks due to the CO₂ emissions associated with its production and potential adverse effects on soil microbiota^15^. Quantitative sustainability assessments are crucial for evaluating the environmental viability of stabilization techniques. Life Cycle Assessment (LCA) studies by Attah et al.^25^ and Ukpata et al.^26^ have established CO₂ emission benchmarks and sustainability indices for soil stabilization methods. For instance, the CO₂ emissions associated with lime stabilization can reach up to 0.75 kg CO₂ per kg of lime produced, whereas integrating recycled plastic waste and natural fibers significantly reduces the carbon footprint, achieving up to 35% lower emissions. Incorporating such quantitative metrics underscores the environmental advantages of the palm fiber–plastic waste combination over conventional lime stabilization.

## Conclusion

This study focused on enhancing the CBR value of fine sand through the incorporation of varying amounts of palm fibers, waste plastic, and quicklime. Laboratory tests were conducted to evaluate the (CBR) values. The conclusions derived from the experimental results are as follows:

- The optimum doses by weight for quicklime, plastic wastes and palm fibers are 5.0%, 0.75% and 1.0% respectively. These doses enhanced the CBR value of fine sand by 195%, 125% and 275% in order.
- Mixing quicklime with palm fibers reduces the CBR value enhancement due to the harmful effect of quicklime on natural organic matter.
- Except for (Lime-Palm fibers) mixture, the CBR enhancements due to using dual additives mixtures are almost the average of enhancements due to using single additive. While the CBR enhancement due to using triple additives is less than their average single enhancements due to Lime-palm fibers effect.
- Although the results showed that mixing the fine sand with palm fibers is the most efficient alternative, but recycling the plastic wastes by mixing them with the palm fibers is more sustainable and environmental friendly alternative.
- The presented enhancement ratios are valid only for the considered materials and must be verified for other type or source of materials.
- For further researches, it is recommended to study the impact of using (Palm fiber-Plastic wastes) dual additives on the pavement economy of highway projects on sand dunes.

This study evaluated the enhancement of fine sand using quicklime, plastic waste, and palm fibers. Optimal dosages were determined, and their individual and combined effects on the CBR were assessed. The most effective enhancement was from palm fiber addition (275%). Dual mixtures offered results near the average of individual contributions, except lime–palm fiber, which underperformed due to chemical incompatibility. While triple mixtures offered moderate improvements, sustainability goals are best met through plastic–palm fiber combinations. This method provides both structural benefit and environmental value, especially for resource-constrained or ecologically sensitive regions. Future studies should explore long-term behavior and environmental impact under variable field conditions.

## Limitations and future work

This study indicates that implementing palm fibers, plastic debris, and quicklime may improve the CBR value of fine sand in the short term, but it doesn’t evaluate how well these additives work over a long time in various environmental conditions. The potential biodegradation of natural palm fibers over time is a significant concern that could lower the stabilized soil’s mechanical stability. Furthermore, nothing is known about the environmental effects of employing plastic waste, including the possibility of microplastic production or the leaching of toxic compounds. In order to confirm the long-term sustainability and safety of the suggested stabilization processes, future research should concentrate on analyzing the materials’ longevity, biodegradability, and environmental impact through extended aging tests, leaching evaluations, and practical field applications.

## Data Availability

All the used data are included in the manuscript.

## References

[1] El-Bosraty, A. H., Ebid, A. M. & Bahr, M. Assessment of efficiencies of different additives to improve CBR value for the highway industry. *Future Eng. J.* **4** (2), 3 (2024). https://digitalcommons.aaru.edu.jo/fej/vol4/iss2/3

[2] Onyelowe, K. C., Ebid, A. M., Nwobia, L. I. & Obianyo, I. I. Shrinkage limit multi–AI–based predictive models for sustainable utilization of activated rice husk Ash for treating expansive pavement subgrade. *Transp. Infrastruct. Geotechnol*. 10.1007/s40515-021-00199-y (2021).

[3] Onyelowe, K. C., Ebid, A. M., Aneke, F. I. & Nwobia, L. I. Different AI predictive models for pavement subgrade stiffness and resilient deformation of geopolymer cement–treated lateritic soil with ordinary cement addition. *Int. J. Pavement Res. Technol.*10.1007/s42947-022-00185-8 (2022).

[4] Onyelowe, K. C. et al. Effect of desiccation on ashcrete (HSDA)-treated soft soil used as flexible pavement foundation: zero carbon stabilizer approach. *Int. J. Low-Carbon Technol.* **17**, 563–570. 10.1093/ijlct/ctac042 (2022).

[5] Huang, S. et al. Plastic waste management strategies and their environmental aspects: A scientometric analysis and comprehensive review. *Int. J. Environ. Res. Public Health*. **19** (8), 4556 (2022). 35457426 10.3390/ijerph19084556 PMC9024989

[6] Abbasi, N. & Mahdieh, M. Improvement of geotechnical properties of silty sand soils using natural Pozzolan and lime. *Int. J. Geo-Eng.* **9**, 1–12 (2018).

[7] Al-Rawas, A. A., Hago, A. W. & Al-Sarmi, H. Effect of lime, cement and Sarooj (artificial pozzolan) on the swelling potential of an expansive soil from Oman. *Build. Environ.* **40** (5), 681–687 (2005).

[8] Baghdadi, Z. A. & Rahman, M. A. The potential of cement kiln dust for the stabilization of Dune sand in highway construction. *Build. Environ.* **25** (4), 285–289 (1990).

[9] Ji-ru, Z. & Xing, C. Stabilization of expansive soil by lime and fly Ash. *J. Wuhan Univ. Technol. Mater. Sci. Ed.* **17** (4), 73–77 (2002).

[10] Elbosraty, A. H., Bahr, M. & Ebid, A. M. Cost optimization for flexible pavement on fine sand improved using palm fibers. *Sci. Rep.* **15**, 17454. 10.1038/s41598-025-02115-7 (2025). 40394116 10.1038/s41598-025-02115-7 PMC12092573

[11] Yusof, Z. M., Zainorabidin, A., Suliman, M. A. O. & Osba, O. E. O. A. Effect of palm Fiber-Hydrated lime composition on the permeability of stabilized sandy soil. *Int. J. Sustain. Constr. Eng. Technol.* **14** (2), 1–6 (2023).

[12] Azadegan, O., Kaffash, A. E., Yaghoubi, M. J. & Pourebrahim, G. R. Swelling, cracking and mechanical characteristics of palm fiber reinforced clay. *Electron. J. Geotech. Eng.* **17** (A), 47–54 (2012).

[13] Kumar, T., Panda, S., Hameed, S. & Maity, J. Behaviour of soil by mixing of plastic strips. *Int. Res. J. Eng. Technol.*, **5**(5). (2018).

[14] Alzaidy, M. N. J. Experimental study for stabilizing clayey soil with eggshell powder and plastic wastes.* IOP Conf. Ser. Mater. Sci. Eng.* 022008. (2019).

[15] Jawad, I. T., Taha, M. R., Majeed, Z. H. & Khan, T. A. Soil stabilization using lime: advantages, disadvantages and proposing a potential alternative. *Res. J. Appl. Sci. Eng. Technol.* **8** (4), 510–520 (2014).

[16] Hejazi, S. M., Sheikhzadeh, M., Abtahi, S. M. & Zadhoush, A. A simple review of soil reinforcement by using natural and synthetic fibers. *Constr. Build. Mater.* **30**, 100–116 (2012).

[17] Brown, S. F. Soil mechanics in pavement engineering. *Géotechnique* **46** (3), 383–426 (1996).

[18] Taskiran, T. Prediction of California bearing ratio (CBR) of fine grained soils by AI methods. *Adv. Eng. Softw.* **41** (6), 886–892 (2010).

[19] Taallah, B. & Guettala, A. The mechanical and physical properties of compressed Earth block stabilized with lime and filled with untreated and alkali-treated date palm fibers. *Constr. Build. Mater.* **104**, 52–62. 10.1016/j.conbuildmat.2015.12.073 (2016).

[20] Olaiya, B. C. et al. An overview of the use and process for enhancing the pozzolanic performance of industrial and agricultural wastes in concrete. *Discov. Appl. Sci.* **7**, 164. 10.1007/s42452-025-06586-1 (2025).

[21] Olaiya, B. C., Lawan, M. M. & Olonade, K. A. Utilization of sawdust composites in construction—a review. *SN Appl. Sci.* **5**, 140. 10.1007/s42452-023-05361-4 (2023).

[22] Olaiya, B. C. et al. Development of sustainable sandcrete bricks using industrial and agricultural waste. *Sci. Rep.* **15**, 17202. 10.1038/s41598-025-02308-0 (2025). 40382438 10.1038/s41598-025-02308-0 PMC12085682

[23] Tiwari, N., Satyam, N. & Puppala, A. J. Strength and durability assessment of expansive soil stabilized with recycled Ash and natural fibers. *Transp. Geotech*. **29**, 100556 (2021).

[24] Haider, A. B., Iravanian, A., Selman, M. H. & Ekinci, A. Using waste PET shreds for soil stabilization: efficiency and durability assessment. *Int. J. Geosynthet. Ground Eng.* **9** (4), 48 (2023).

[25] Attah, I. C., Alaneme, G. U., Fadugba, O. G. & Olaiya, B. C. Sustainability measurement of developed concrete by incorporating solid waste. In* Recent Developments and Innovations in the Sustainable Production of Concrete*. Woodhead Publishing, 333–347. 10.1016/B978-0-443-23895-6.00012-1 (2025).

[26] Ukpata, J. O. et al. Effects of aggregate sizes on the performance of laterized concrete. *Sci. Rep.* **14**, 448. 10.1038/s41598-023-50998-1 (2024). 38172194 10.1038/s41598-023-50998-1 PMC10764962

